# A local-saturation-and-delay MRI method for evaluation of red blood cells aggregation *in vivo* for tumor-bearing or drug-used rats

**DOI:** 10.3389/fbioe.2023.1111840

**Published:** 2023-01-17

**Authors:** Haiwei Shan, Lei Shi, Shuang Liu, Yaping Yuan, Hongchuang Li, Shizhen Chen, Xin Zhou

**Affiliations:** ^1^ State Key Laboratory of Magnetic Resonance and Atomic and Molecular Physics, Innovation Academy for Precision Measurement Science and Technology, Chinese Academy of Science—Wuhan National Laboratory for Optoelectronics, Wuhan, China; ^2^ University of Chinese Academy of Sciences, Beijing, China; ^3^ Department of Pediatrics, Affiliated Hospital of Changchun University of Traditional Chinese Medicine, Changchun, China

**Keywords:** magnetic resonance imaging, local-saturation-and-delay imaging, hyperviscosity syndrome, sprague-dawley rats, whole blood relative viscosity

## Abstract

Hyperviscosity syndrome (HVS) is a combination of clinical signs and symptoms related to increased blood viscosity. HVS can increase the thrombotic risk by causing a major disturbance to the blood flow, which is usually found in the advanced stages of the tumor. Moreover, some of the drugs used in chemotherapy, such as 5-fluorouracil and erythropoietin, are also capable of causing HVS through their respective pathways. Clinically, the viscosity of a patient’s blood sample is measured by a rotary rheometer to estimate the risk of hyperviscosity syndrome. However, the measurement of blood viscosity *in vitro* is easily affected by storage time, storage environment, and anticoagulants. In addition, the fluid conditions in the rheometer are quite different from those in natural blood vessels, making this method inappropriate for evaluating blood viscosity and its effects *in vivo* under physiological condition. Herein, we presented a novel magnetic resonance imaging method called local-saturation-and-delay imaging (LSDI). The radial distributions of flow velocity measured by LSDI are consistent with the Ultrasonic (US) method (Spearman correlation coefficient r = 0.990). But the result of LSDI is more stable than US (*p* < 0.0001). With the LSDI method, we can directly measure the radial distribution of diastolic flow velocity, and further use these data to calculate the whole blood relative viscosity (WBRV) and erythrocyte aggregation trend. It was a strong correlation between the results measured by LSDI and rotary rheometer in the group of rats given erythropoietin. Furthermore, experimental results in glioma rats indicate that LSDI is equivalent to a rheometer as a method for predicting the risk of hyperviscosity syndrome. Therefore, LSDI, as a non-invasive method, can effectively follow the changes in WBRV in rats and avoid the effect of blood sampling during the experiment on the results. In conclusion, LSDI is expected to become a novel method for real-time *in vivo* recognition of the cancer progression and the influence of drugs on blood viscosity and RBC aggregation.

## 1 Introduction

Red blood cell (RBC) aggregation is a reversible phenomenon that occurs at a low shear rate. Some factors such as diabetes (Jax et al., 2009), cancer (Lewis et al., 2011), inflammation (Zimran et al., 2004), and certain drugs (Zbinden, 1976; Muravyov et al., 2010; Yuval et al., 2013; Girolami et al., 2017; Kalaska et al., 2022) can change the composition of the blood and properties of the RBCs, leading to hyper aggregation of cellular components in the blood. It is the cause of perfusion injury and impaired tissue oxygenation (Ostergaard et al., 2014).

In clinical practice, rotary rheometers are commonly used to estimate the aggregation of RBCs by measuring the viscosity of the whole blood and plasma in a series of shear rates. When whole blood viscosity is measured with a rheometer, the blood sample flows inside the container as a pattern of uniform shear rate (Couette flow). For blood, a non-Newtonian fluid, sufficiently stable results can be obtained with a rotary rheometer. But the flow of blood *in vivo* is Poiseuille flow, which is quite different from Couette flow (Govindarajan and Sahu, 2014). Therefore, it is not appropriate to use a rotary rheometer to represent the fluidic conditions in a particular part of the body. In addition, there are some other factors with hemorheological testing *in vitro*, such as anticoagulants and storage conditions (Marcel et al., 2021).

RBCs migrate toward the center due to the properties of their membranes. And this phenomenon leads to a type of two-phase blood flow eventually (Goldsmith, 1971; Secomb, 2017). These contribute to the fact that blood shows non-Newtonian fluidity at the macroscopic scale which was discovered by first Fahraeus and Lindqvist (1931). The two-phase flow consists of a cell-free layer next to the vessel wall and a core region at the center. The RBCs flow with relatively higher velocity in the core region, leaving a pure plasma region in cell-free layer with a lower velocity close to the vessel wall. This model has been confirmed by experiments *in vitro* and in microvascular (Pries et al., 1992; Kim et al., 2009). Measurements in larger vessels also show that the flow profile of blood is blunter than Newtonian fluid (Shehada et al., 1994; Ade et al., 2012). Moreover, Cherry et al. found that vessel diameter affects the viscosity properties of blood in the core region. And the core region of blood flow conforms to the behavior of Newtonian fluid when the diameter is smaller than 3 mm (Pries et al., 1992; Cherry and Eaton, 2013).

**FIGURE 1:**
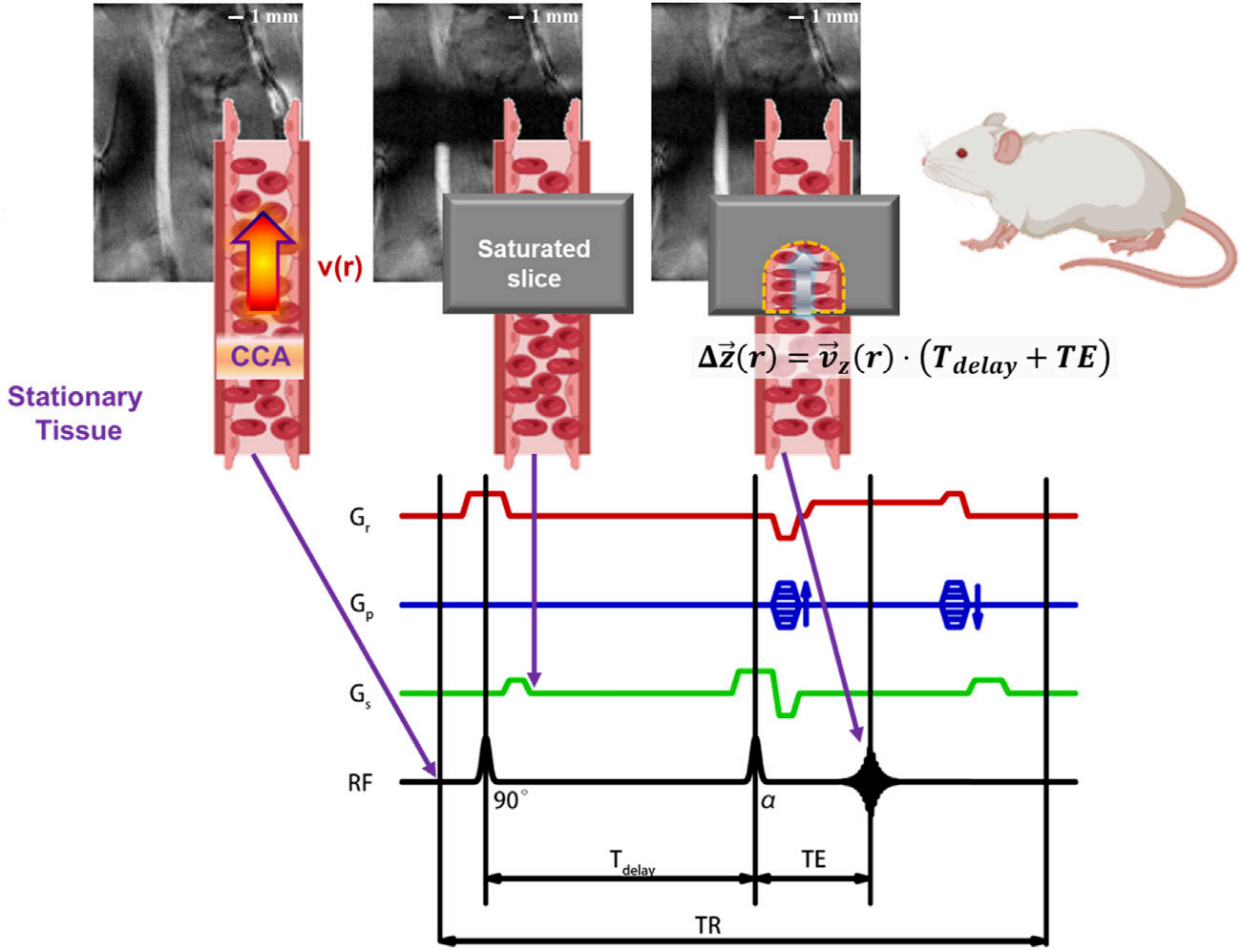
Schematic diagram of LSDI applied to the left common carotid artery of SD rats. The essence of the LSDI method is that the partially overlap of TOF-MRA and T_1_-weight FLASH in the field of view, and the path of blood flow can be visualized by adjusting the delay time. This method performs a T_1_-weight FLASH scan outside the saturated slice to observe the vascular geometry upstream and downstream of the region of interest and ensures that the saturated slice is perpendicular to the blood vessel. At the same time, a TOF scan with a specified delay time is performed in the saturated slice to observe the displacement of the blood flow at different radial positions and eventually obtain the flow velocity distribution.

Magnetic resonance imaging (MRI) is one of the commonly used blood flow imaging techniques in the clinic. It has a greater penetration depth than optical imaging and no invasive or ionizing radiation. MRI is suitable for imaging the common carotid artery (CCA) and other deep vessels. Here, an MRI method named local-saturation-and-delay imaging (LSDI) has been developed, as shown in Figure 1. The LSDI method has three steps. In the first step, the subject is saturated in a local space of 5 mm thickness. The saturated slice is perpendicular to the blood flow direction, and the transverse magnetization vector in this region is eliminated by a spoiled gradient. The second step is the no-action delay. A delay time (T_delay_) of 8–18 ms is recommended for Sprague-Dawley rats. Since the delay time is much shorter than the longitudinal relaxation time of the blood, there is a significant difference in the signal intensity between inside and outside the saturated slice. During this period, a portion of blood inflow to the saturated slice from outside and recovers the longitudinal magnetization vector of the affected region in the saturated slice. In the third step, the signal distribution in the slice containing the vessel is read out by the T_1_-weight FLASH method. The edges of the saturated section will sink inwards in the resulting image due to the inflow of blood in the T_delay_ and echo time (TE). Since blood flows laminar through the vessel, the shape of the sink is related to the radial distribution of the blood flow velocity. In summary, LSDI is only sensitive to the flow in T_delay_ and TE. We refer to this period as the flow-sensitive window.

In this study, we use a two-phase model to analyze the image results based on the distribution of blood flow velocities obtained by the LSDI method, enabling *in vivo* non-invasive measurements of the viscosity properties of blood vessels. The LSDI method was compared with the results of ultrasonic (US) and rotary rheometers to confirm the effectiveness of LSDI in the measurement of radial velocity distribution and whole blood relative viscosity (WBRV), respectively. In addition, LSDI results in glioma rats demonstrate the effectiveness of this novel method in assessing the risk of hyperviscosity syndrome in cancerous individuals. This study provides a method for tracking the relative viscosity of whole blood in experimental animals (e.g., rats). And the development of such methods is conducive to the study of blood viscosity in animal models related to diseases or drugs.

## 2 Materials and methods

### 2.1 Animal experiments

All animal protocols were approved by the Committee on Animal Research and Ethics of APM, CAS. Eight-week-old male Sprague-Dawley rats were used. Animals had free access to food and water with a natural light/dark cycle at 23°C. After one-week of adaptive feeding, the animals were divided into NC group and EPO group randomly (5 of each). High dose human recombinant erythropoietin (rhEPO) 2000 U/Kg (Kexing Biopharm Co., Ltd.) was employed for injections intraperitoneally to rats in EPO group on days 1, 3, 6, and 9, lasting for 11 consecutive days. The control group was given normal saline in the same amount. In addition, MRI and US tests were performed on days 0, 5, and 11 for both groups of rats. Immediately after the MRI and US tests, all the rats were euthanized and whole blood was taken for hemorheology measurements.

In addition to the above two groups of rats, a group of glioma model rats (C6 group, SD rat, n = 5) was set up. Rats in the C6 group were accommodated under the same conditions as those in the NC and EPO groups and fed the same diet. The tumor implantation protocol followed by Karmakar et al. (2007). C6 cells were implanted into C6 group rats by intracerebral implantation with a 10^6^ in 5 μL PBS concentration. MRI was performed at T_2_-weighted imaging (T_2_WI) and LSDI on day 11 after implantation. Whole blood samples were taken and evaluated by the same protocol as the EPO group.

### 2.2 Local-saturation-and-delay imaging (LSDI) protocol

All MRI scans were carried out on a Biospec 70/20 (Bruker BioSpin, Billerica, MA, United States) with a 30 mm diameter 300 MHz surface RF coil and ParaVision version 5.1. For imaging, all the rats were anesthetized with isoflurane mixed with oxygen. The surface coil is placed over the rat’s neck. The region of interest in the left CCA was identified by multilayer MRI of the neck.

The imaging parameters were: Field of view (FOV): 20 × 14 × 2 (slice thick) mm^3^, Repetition time (TR)/Echo time (TE): 100 ms/2.428 ms, Flip Angle: 40°, Number of Average: 20 times, matrix: 128 × 96, saturated slice thick: 5 mm. T_delay_ was chosen between 8 ms and 18 ms, depending on the blood flow velocity, to ensure that the blood flow distance was sufficient but not beyond the saturated slice. Since the readout direction is perpendicular to the saturated slice in LSDI. We adjusted the field of view so that the reading gradient direction was parallel to the direction of flow. With these parameters, the spatial resolution is 0.156 × 0.146 mm^2^ and the total scan time is 3 min 12 s.

Gliomas were observed in the C6 group by T_2_WI with the following parameters: Method: MSME, FOV: 30 × 30 × 13 (slice thick) mm^3^, TR: 2500 ms, TE: 15, 30, 45, 60, 75, 90, 105, 120, 135, and 150 ms, matrix: 128 × 128 × 9 (number of slices). The total scan time under these parameters was 5 min 20 s.

### 2.3 Whole blood relative viscosity (WBRV) calculated by the two-phase model

The inhomogeneous spatial distribution of RBCs in blood flow leads to an unequal radial distribution of viscosity. Since this method does not investigate the traces of individual RBCs or RBC rouleaux, several approximations can be applied to make the calculation brief. According to the two-phase model, the core region and the cell-free layer can be considered as two phases with different viscosities. This approximation was first proposed by Vand and verified in the pipe with a diameter from 30 μm to 1,000 μm (Vand, 1948; Pries et al., 1992), and the specific form is described in Eq. 1:

$$\begin{array}{c} \mu \left(r\right)=\left\{\begin{array}{l} \mu_{c}\;0\leq r\leq {\lambda r}_{vessel}\\ \mu_{p}\;{\;\lambda r}_{vessel}<r<r_{vessel} \end{array}\right. \end{array}$$ (1)

Where r_vessel_ is the inner radius of the vessel, μ_c_ is the viscosity of core region, μ_p_ is the viscosity of plasma, λ = 1-δ/r_vessel_ is the ratio of core region’s radius and inner radius of vessel, δ is the width of the cell-free layer. The variation of the vessel diameter due to pressure fluctuations in the diastole phase is much smaller than its mean diameter. In this case, the vessel can be approximated as a rigid tube. The flow direction is defined as the *Z*-axis, and the axial component of Navier-Stokes Eq. 2 equations in cylindrical coordinates read

$$\begin{array}{c} -\frac{\partial p}{\partial z}+\mu \left(\frac{1}{r}\frac{\partial }{\partial r}\left(r\frac{\partial v_{z}}{\partial r}\right)+\frac{1}{r^{2}}\frac{\partial^{2}v_{z}}{\partial \theta^{2}}+\frac{\partial^{2}v_{z}}{\partial z^{2}}\right)=\rho \left(\frac{\partial v_{z}}{\partial t}+v_{r}\frac{\partial v_{z}}{\partial r}+\frac{v_{\theta }}{r}\frac{\partial v_{z}}{\partial \theta }+v_{z}\frac{\partial v_{z}}{\partial z}\right) \end{array}$$ (2)

together with continuity Eq. 3

$$\begin{array}{c} \frac{\partial v_{z}}{\partial z}=0 \end{array}$$ (3)

Where r, θ, z are the three dimensions of cylindrical coordinates, t is the time dimension, ρ is the density, μ is the viscosity of the fluid, ∂p/∂z is the pressure drop in the flow, and v_z_, v_r,_ v_θ_ are the velocities in all three dimensions of the flow. The cross-section of the CCA can be viewed as a circle, so the blood flow through it should be symmetric. Eq. 2 reduces to

$$\begin{array}{c} -\frac{\partial p}{\partial z}=\rho \frac{\partial v_{z}}{\partial t}-\mu \left[\frac{1}{r}\frac{\partial }{\partial r}\left(r\frac{\partial v_{z}}{\partial r}\right)\right] \end{array}$$ (4)

During diastole, blood flow in the CCA is slow and steady. Therefore, the effect of velocity and pressure fluctuations can be suppressed by sampling low velocity blood flow signals selectively. In this case, the partial derivative of diastolic velocity to time is negligible (Maya et al., 2014). The pressure can also be considered as a time-independent variable. Eq. 4 can be regarded as Stokes flow

$$\begin{array}{c} \frac{dp}{dz}=\mu \left(r\right)\left[\frac{1}{r}\frac{d}{dr}\left(r\frac{dv_{z}}{dr}\right)\right] \end{array}$$ (5)

Notably, the value of μ(r) has been determined in Eq. 1, and analytic expression of velocity profile Eq. 8 can be solved simultaneously by Eq. 3, non-slip boundary condition Eq. 6, continuity of velocity distribution Eq. 7, and the simplified Navier-Stokes Eq. 5:

$$\begin{array}{c} v_{z}\left(r_{vessel}\right)=0 \end{array}$$ (6)

$$\begin{array}{c} \lim_{r\to {{\lambda r}_{vessel}}^{-}}v_{z}\left(r\right)=\lim_{r\to {{\lambda r}_{vessel}}^{+}}v_{z}\left(r\right) \end{array}$$ (7)

$$\begin{array}{c} v_{z}\left(r\right)=\left\{\begin{array}{c} \frac{1}{4\mu_{p}}A\left(r_{vessel}^{2}-r^{2}\right)\;\lambda r_{vessel}<r<r_{vessel}\\ \frac{Ar_{vessel}^{2}}{4}\left[\frac{\lambda^{2}-{\left(\frac{r}{r_{vessel}}\right)}^{2}}{\mu_{c}}-\frac{1-\lambda^{2}}{\mu_{p}}\right]\;0\leq r\leq \lambda r_{vessel} \end{array}\right. \end{array}$$ (8)

Where A = dp/dz. As Eq. 8, blood flow velocity profile is present as a two-segment parabola bounded by r = λr_vessel_. The ratio of the quadratic coefficients of the two curves is given by:

$$\begin{array}{c} \frac{-\frac{1}{4\mu_{p}}A}{-\frac{1}{4\mu_{c}}A}=\frac{\mu_{c}}{\mu_{p}} \end{array}$$ (9)

Because taking blood means sampling the core region. The viscosity of core region μ_c_ can be regarded as whole blood viscosity (WBV). That means the ratio in Eq. 9 has a direct correlation with WBRV (η_r_). The value of η_r_ shows the level of blood cell aggregation and deformability during flow (Beilin et al., 2006; Wang et al., 2018). In this way, we transform the problem of measuring viscosity into a process of measuring the radial velocity distribution, also known as the velocity profile.

### 2.4 Ultrasonic protocol

The US scanning protocol is performed with the VisualSonic Vevo2100 Ultrasonic scanner (FUJIFILM VisualSonics inc.). The rat was anesthetized with isoflurane and the left CCA was scanned with an MS-400 probe. More than 10 cardiac cycles were recorded in both pulsed and color Doppler modes. For the two modes of scanning, the probe insonation angle was maintained at 15° and the frequency used was 24 MHz.

The vascular resistance index (VRI) was calculated according to standard formulae as Eq. 10.

$$\begin{array}{c} VRI=\frac{PSV-EDV}{PSV} \end{array}$$ (10)

Where PSV = peak systolic velocity, EDV = end-diastolic velocity. An abnormal elevation of the VRI indicates blood flow stasis, which is an important indicator of vascular stenosis and thrombosis as measured by the pulsed Doppler mode.

### 2.5 Hemorheological tests

After MRI and US scan on day 11, the rats in the NC and EPO groups were euthanized. Arterial blood was immediately collected from all the rats. Approximately 2 mL of blood was transferred into a tube containing sodium citrate and the remainder into a heparin tube. All the tubes were numbered and sent to Kindstar Globalgene Technology, Inc. for hemorheological testing. A similar protocol was used for rats in the C6 group.

### 2.6 Local-saturation-and-delay image processing

**FIGURE 2:**
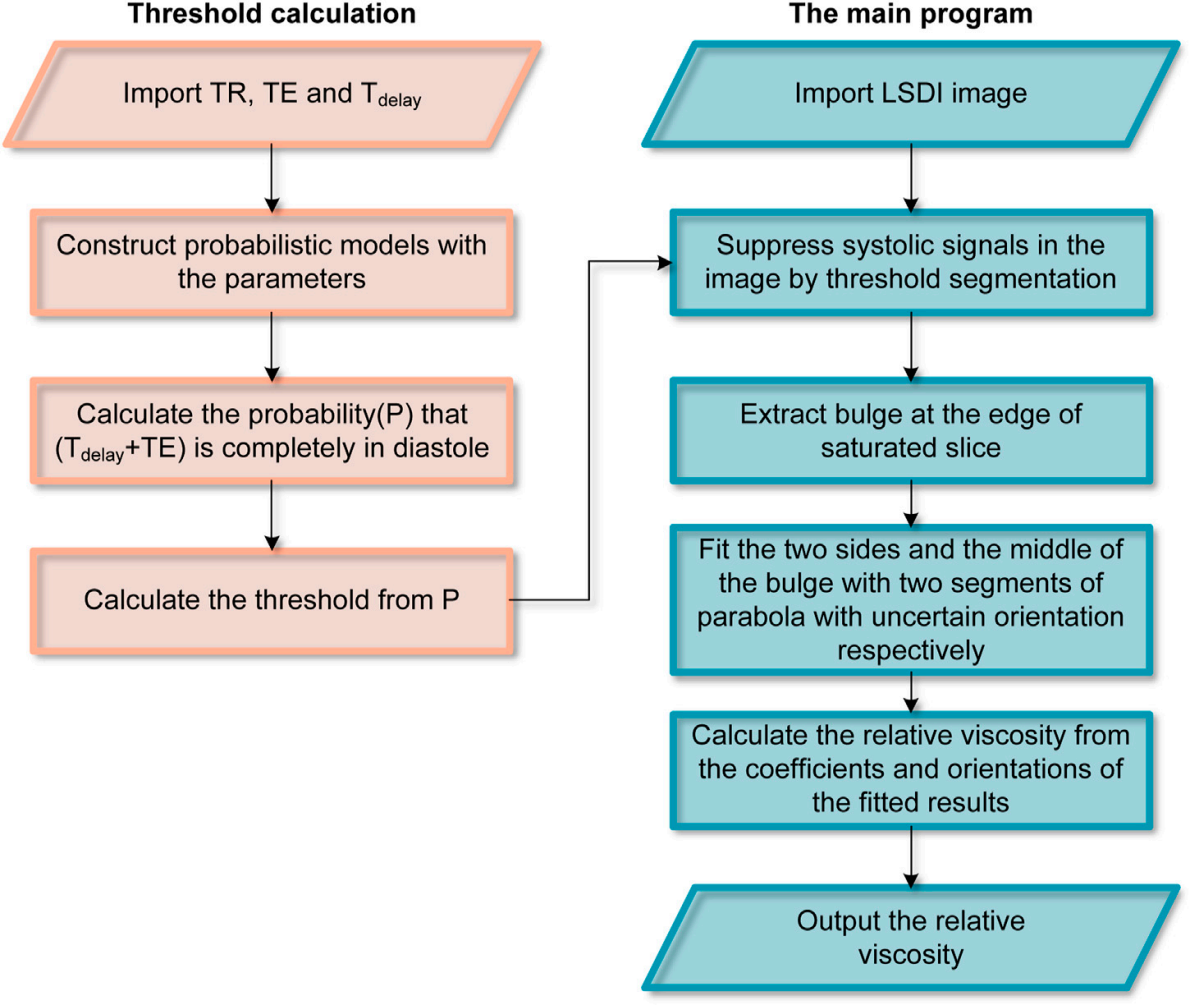
Workflow for extracting WBRV from LSDI images.

LSDI image processing is carried out with a self-built Python program. Figure 2 shows the workflow of image processing. The images were cropped to remove regions that did not contain blood flow features to reduce the computational burden of the procedure. The images are then segmented by thresholding the image intensities to suppress systolic blood flow information. The threshold is calculated from the parameters used by LSDI. 0.42 can be used as a threshold at TR = 100 ms, TE = 2.428 ms and T_delay_ = 17.572 ms. Finally, according to the two-phase model, the edge points in the saturated slice are divided into two groups. Based on the coordinates of these points, the expressions of two parabolas and rotation correction angles are calculated. Bicubic interpolation is used to obtain more reference points during LSDI image processing.

## 3 Results

### 3.1 LSDI results of NC group and EPO group

**FIGURE 3:**
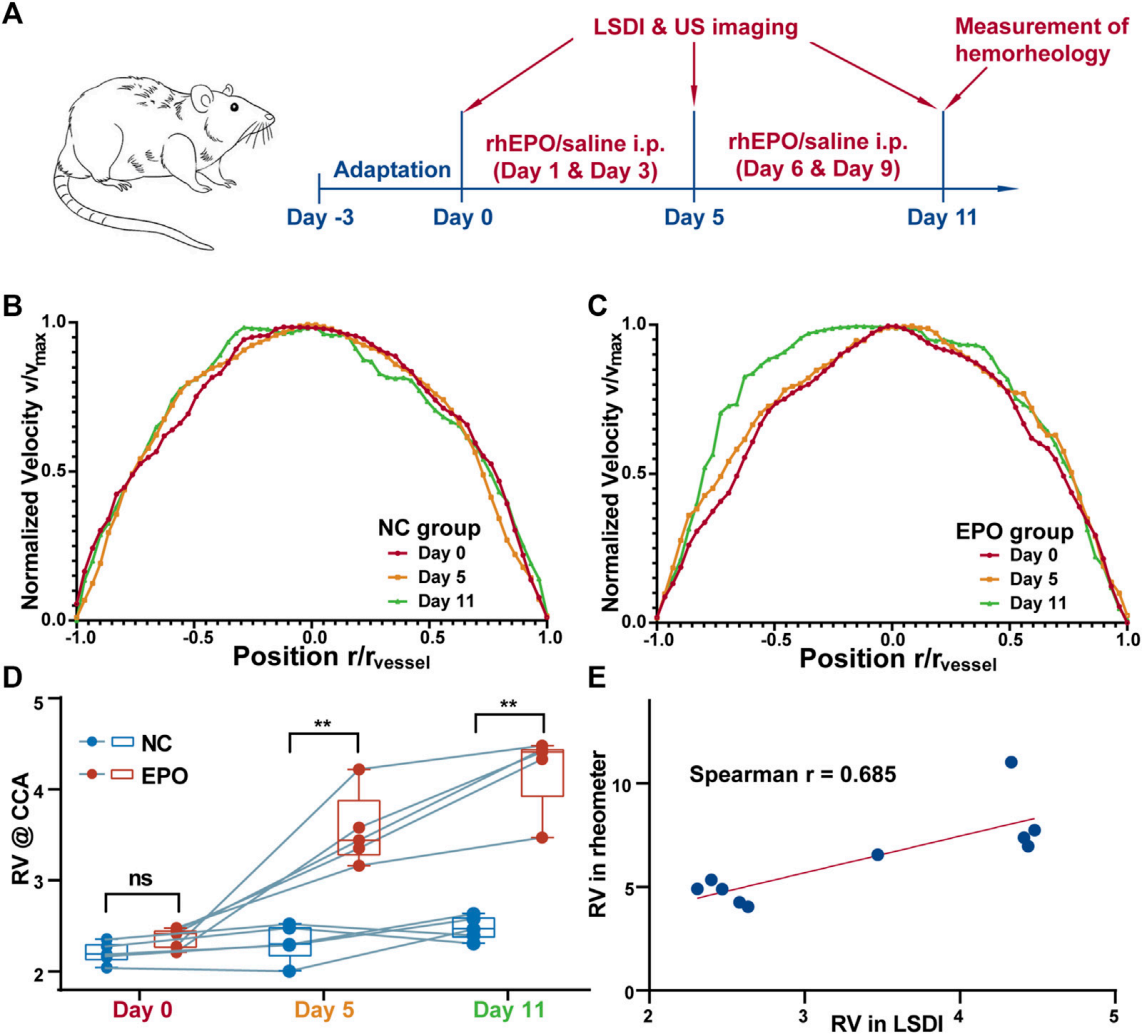
The timeline of the experiments **(A)**. Normalized blood flow velocity profiles (measured by LSDI) of control group **(B)** and EPO group **(C)**. The profiles of the EPO group showed obvious passivation on day 11. The WBRV (measured by LSDI) of NC and EPO group of animals **(D)**. The WBRV in EPO group increased significantly (**: *p* < 0.01). The correlation between the WBRV of the two group measured by LSDI and rheometer on day 11 **(E)**. There is a strong correlation between the results measured by two methods. (Spearman r = 0.685, *p* < 0.05).

The LSDI results for all SD rats on days 0, 5 and 11 were processed as described above to obtain the radial distribution of streamwise velocities for the images on the three days. Typical individual results for the control and EPO groups are shown in Figures 3B, C, respectively. Figure 3B shows the distribution for one rat in the NC group, and there is no significant difference among the results. Figure 3C shows the same distribution for another rat in the EPO group. For the rats in the EPO group, the distribution had changed on day 5. The absolute value of the quadratic coefficient decreases in the core region and increases near the vessel wall. On day 11, the change was more marked. The quantification results for the WBRV are shown in Figure 3D. The WBRV in the EPO group was significantly higher than that in the control group on day 5 and day 11 (*p* < 0.005, EPO group *η*_*r*_ = 4.23 ± 0.72, NC group *η*_*r*_ = 2.48 ± 0.51 on day 11).

### 3.2 US experiments of NC group and EPO group

The results for the pulsed Doppler mode are shown in Supplementary Figure S1. The cardiac cycle can be divided into systolic and diastolic phases based on the end-diastolic flow rate. The ratio of systolic to diastolic length was 2:3. The vascular resistance index (VRI) of the two groups of rats were calculated by Eq. 10. The results are shown in Supplementary Figure S2. There was no significant difference between the NC and EPO groups.

**FIGURE 4:**
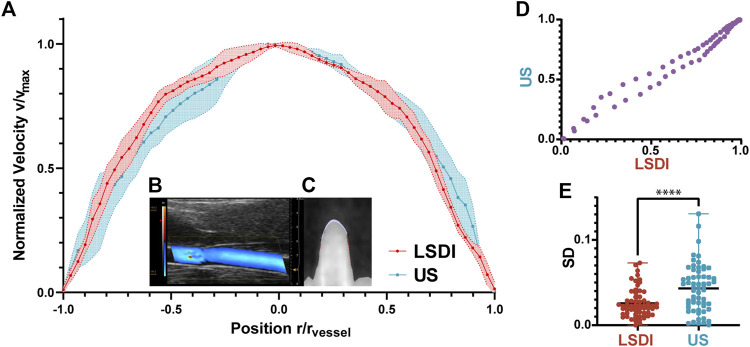
Normalized blood flow velocity profiles measured by LSDI and ultrasonic **(A)**. Ultrasonic imaging of blood flow in CCA scanned by color Doppler flow imaging mode **(B)**. LSDI imaging of blood flow in CCA **(C)**. The correlation between the two groups of datapoints measured by LSDI and US **(D)**. The velocity profiles obtained by the two methods are consistent (Spearman r = 0.990, *p* < 0.0001). Standard deviation distribution of datapoints in the two methods **(E)**, ****: *p* < 0.0001).

In Figure 4A, we compare the radial profiles of the flow velocities obtained with LSDI and color Doppler ultrasonic mode. The results of color Doppler were obtained in diastolic phase from the rats in NC group (as Figure 4B). LSDI results were extracted from threshold segmented images to suppress systolic signal (as Figure 4C). The consistency between the results of the LSDI and color Doppler is shown in Figure 4D (Spearman correlation coefficient r = 0.990, *p* < 0.0001). The standard deviation distribution for each data point in both methods is shown in Figure 4E. LSDI results are relatively more stable than color Doppler imaging (*p* < 0.001).

### 3.3 Hemorheological test results of NC group and EPO group

The results for hemorheological tests are shown in Table 1. The WBRV of the EPO group increased significantly under high shear rate (200 s^−1^). There were also significant differences in erythrocyte rigidity index (RI). Figure 3E shows that both LSDI and hemorheological tests were able to distinguish the difference in WBRV between the two groups of rats. Between the two methods, the Spearman correlation coefficient is 0.685.

**TABLE 1:** Results of hemorheological tests.

Rat no.	NC 1	NC 2	NC 3	NC 4	NC 5	EPO 6	EPO 7	EPO 8	EPO 9	EPO 10	C6 11	C6 12	C6 13
WBV (mPa·s)													
1 s^−1^^*, ^+^^	36.49	25.66	28.26	40.61	43.06	49.72	41.38	44.14	43.02	40.80	48.15	52.52	46.81
50 s^−1^^****, ^+^^	5.96	6.59	5.96	6.87	6.83	9.58	8.68	12.45	9.14	10.59	8.04	8.66	7.85
200 s^−1^^***, ^+^^	4.60	5.58	4.86	5.34	5.23	7.67	7.07	10.70	7.46	8.98	6.41	6.70	6.18
WBRV													
1 s^−1^	33.79	22.51	23.55	37.26	43.94	47.81	38.31	45.51	40.21	35.17	41.15	42.35	39.01
200 s^−1^^**^	4.26	4.89	4.05	4.90	5.34	7.38	6.55	11.03	6.97	7.74	5.47	5.40	5.15
Other													
PV (mPa·s)	1.08	1.14	1.20	1.09	0.98	1.04	1.08	0.97	1.07	1.16	1.17	1.24	1.20
HCT	0.49	0.45	0.52	0.53	0.52	0.53	0.50	0.57	0.53	0.55	0.59	0.46	0.46
AI	7.93	4.60	5.81	7.60	8.23	6.48	5.85	4.13	5.77	4.54	7.51	7.84	7.57
RI ^**^	6.65	8.65	5.87	7.36	8.34	12.03	11.09	17.60	11.27	12.26	7.58	9.56	9.02

Differences between the NC and EPO groups ^*^ *p* < 0.05, ^**^ *p* < 0.01, ^***^ *p* < 0.001. Differences between the NC and C6 groups ^+^ *p* < 0.05.

WBV, whole blood viscosity, PV; plasma viscosity, WBRV, whole blood relative viscosity; HCT, hematocrit; AI, aggregation index; RI, rigid index.

### 3.4 LSDI and hemorheological test results of glioma rats

**FIGURE 5:**
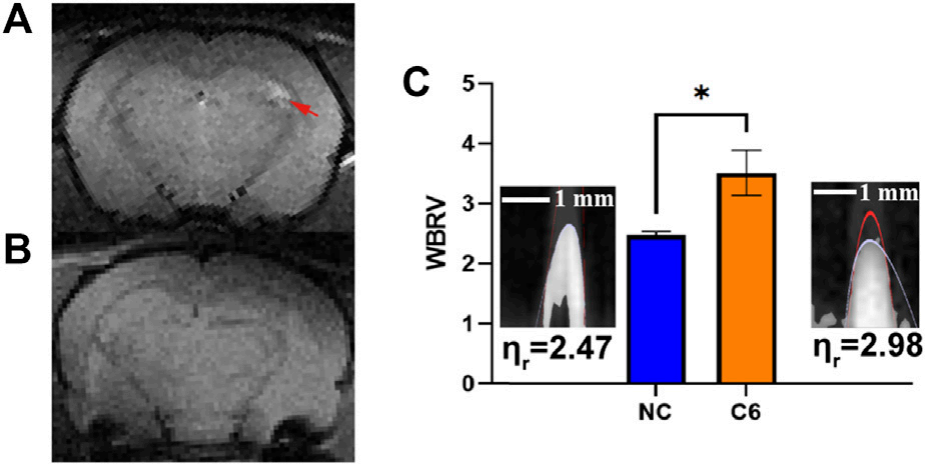
T_2_WI for the brain in C6 rat **(A)** and NC rat **(B)**. The rats’ WBRV (measured in the left common carotid artery by LSDI) of NC and C6 groups **(C)**, ^*^: *p* < 0.05).

The results of hemorheological tests for the C6 group are shown in Table 1. The results of two rats were invalidated due to premature death during blood collection. Prior to measuring the WBRV with LSDI, brain imaging of C6 rats as shown in Figure 5A was performed using T_2_WI to confirm the glioma volume. Figure 5B showed the image of the same part of the NC rats. Figure 5C shows the difference in WBRV on day 11 between the NC and C6 groups of rats (*p* < 0.05). The results indicate that LSDI and rheometer are equally effective in assessing the WBRV of the carcinoma rat model.

## 4 Discussion

### 4.1 Effectiveness of LSDI in WBRV measurement

In this study, we developed and performed a non-invasive MRI-based protocol to measure WBRV in rats. This protocol is suitable for the rats that require follow-up in hemorheology. This is an *in vivo* method to calculate the viscosity properties from the velocity profile backwards. We first obtained a stable and visualized blood flow profile using LSDI imaging. We then summarize a method to calculate the WBRV from the velocity profile in combination with the two-phase model proposed by previous researchers. The WBRV obtained in this way is strongly correlated with the results of hemorheology tests commonly used in clinical practice. Both can distinguish between cancer or drug-induced increases in WBRV. However, the method without blood collection is useful in observing how rats respond to withdrawal or treatment.

### 4.2 Calculation of segmentation threshold in image processing

According to Eq. 4, fluctuations in blood vessels may affect the shape of the velocity profile. This phenomenon has also been shown in previous studies (Paeng et al., 2004). Fortunately, blood flow in the vessels is generally laminar. Thus, in LSDI images, the signal in the high-velocity systolic period can always cover the signal in the diastolic period. Therefore, threshold segmentation can suppress the boundary blurring and distortion caused by systolic period.

Here, the threshold is determined by the LSDI parameters and the cardiac cycle. When the repetition time is significantly different from the length of the cardiac cycle, the LSDI imaging result is a weighted average of the diastolic and systolic signals with their sampling probabilities. If different blood flow velocities are displayed with the same signal amplitude in the image, the threshold for segmentation is given by Eq. 11.

$$\begin{array}{c} P=1-\frac{T_{dia}-\tau }{T_{C}}\;\tau \in \left[10ms,\;20ms\right] \end{array}$$ (11)

Where P is the probability that the flow-sensitive window is not completely included in the diastolic period, which is also the segmentation threshold based on the above assumptions. T_C_ is the cardiac cycle and T_dia_ is the length of diastole in one cycle. τ = T_delay_ + TE is the window length of flow sensitivity. The systolic high-flow signal in the images is relatively weak due to the phase loss caused by the flow. Summary, the actual segmentation threshold used is about 80% of P to prevent excessive filtering of signals near the vessel wall.

### 4.3 WBRV in rats

For more details on the rats in EPO group, we refer to the previous work of Li et al. (2022). During administration, rats in the EPO and NC groups were scanned for WBRV using LSDI. Rats in the EPO group showed a significant increase in WBRV on day 5 and further increases from day 5 to day 11. Our data suggest that rhEPO causes an increase in blood viscosity significantly earlier than the end of administration in rats in other studies (Wilcox et al., 1993; Li et al., 2022). This is due to the different purpose of the study. rhEPO has a more direct effect on WBRV than the indicators of interest in other studies.

The LSDI results of the C6 group also showed a significant increase compared to the NC group. This conclusion is consistent with the hemorheological test results in Table 1, and consistent with other studies (Cai et al., 2014).

### 4.4 Transformation of fluid properties with the WBRV

In previous studies, researchers tended to treat the core region in the two-phase model as Newtonian (Lopes et al., 2020) or Casson fluids (Rana and Murthy, 2016; Roy and Shaw, 2021). The main difference between the two types of fluid is whether there is another region at the axis of the core region with almost no shear rate. As shown in Figure 3B, no such region is present in the blood flow of the NC rats. However, with the injection of rhEPO, the LSDI results on day 11 showed a rather low shear rate region at the vessel axis (Figure 3C). This suggests that, in addition to the effect of vessel diameter, changes in hemorheological properties can switch the nature of the core region between the two types of fluids. Therefore, this property should be considered in the selection of mathematical models for blood flow in simulations, rather than simply based on vessel diameters.

### 4.5 Limitations of the LSDI in spatial resolution

The spatial resolution of the LSDI method is larger than the thickness of the cell-free layer estimated *in vitro* by Pries et al. (1992). In the core region of the CCA, however, LSDI can obtain blood velocity profiles that are flatter than those of a Newtonian fluid. Furthermore, from raw MRI images (Supplementary Figure S3), it is observed that the signal near the vessel wall is decreasing along the flow direction. From this it may be ascertained that there is a lubricating layer of a certain width near the vessel wall.

In addition, as shown in Figure 3, the WBRV results from LSDI are lower than those from rheometer measurements. There are three reasons for this: First, the width of the cell-free layer is overestimated due to the lack of spatial resolution. Second, tropism of the red blood cell flow causes the hematocrit of the blood sample to be higher than *in vivo*. Third, the effect of anticoagulants in blood samples. Nevertheless, we still demonstrate the ability of the LSDI method to distinguish individuals with abnormal WBRV. There is also a strong correlation between LSDI and rheometers.

## 5 Conclusion

In this study, we developed a method called LSDI to measure the blood flow profile and assess the WBRV in the CCA of SD rats without blood sampling. The LSDI protocol was able to anesthetize rats and capture three images in 15 min. There was a strong positive correlation between LSDI and hemorheology test (Spearman correlation coefficient r = 0.685). And the measurements of the velocity profile by LSDI are more stable than those by color Doppler ultrasonic imaging.

The LSDI method caused no harm to the rats. Therefore, it is an ideal tool for tracking changes in hemorheological properties in preclinical studies. It is useful to study the viscosity properties of blood at a particular site or at a particular stage, to analyze the relationship between changes in properties of hemorheology and disease or drugs, and to assess the risk of hyperviscosity syndrome in carcinoma models.

## Data Availability

The raw data supporting the conclusions of this article will be made available by the authors, without undue reservation.
