# Supplementary material for: A local-saturation-and-delay MRI method for evaluation of red blood cells aggregation in vivo for tumor-bearing or drug-used rats
Source: Front Bioeng Biotechnol. 2023 Jan 17;11:1111840. doi: 10.3389/fbioe.2023.1111840 (PMC9887193; doi:10.3389/fbioe.2023.1111840)
Supplement: Supplementary file 1 [file Table1.DOCX]

Supplementary Material


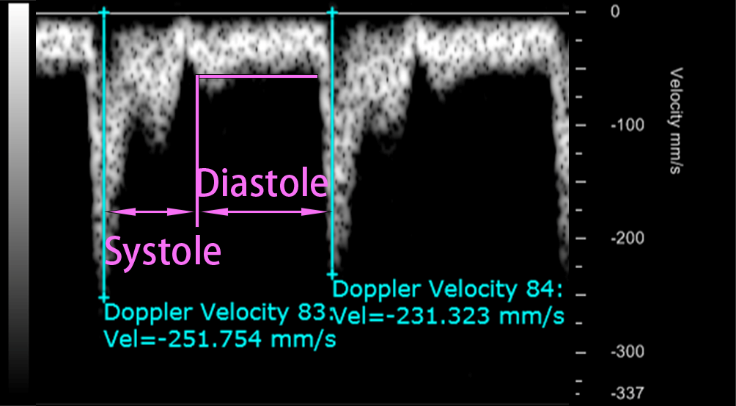


**Supplementary Figure S1.** Doppler ultrasonic spectrum of common carotid artery in NC group. The systolic/diastolic ratio of the common carotid artery in rats was about 2:3


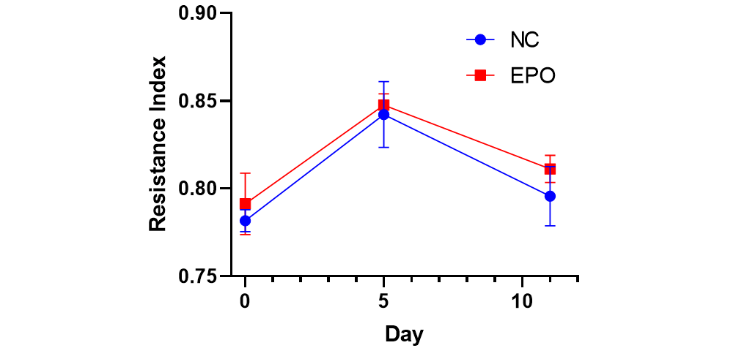


**Supplementary Figure S2.** Changes of vascular resistance index in rats. There was no significant difference in vascular resistance index between the two groups during the rhEPO administration.


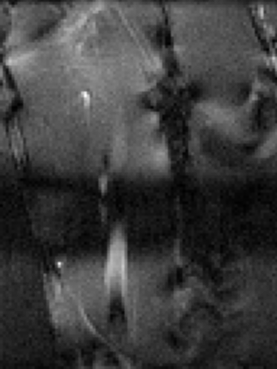


**Supplementary Figure S3.** Raw image of LSDI in left CCA of SD rat. The inflow pattern of the vessel shows a large slip near the wall, which is consistent with the conclusion of the two-phase model in the case of resolution larger than the thickness of the cell-free layer.
